# *KIR* and *HLA* Genotypes Implicated in Reduced Killer Lymphocytes Immunity Are Associated with Vogt-Koyanagi-Harada Disease

**DOI:** 10.1371/journal.pone.0160392

**Published:** 2016-08-04

**Authors:** Ralph D. Levinson, Madeline Yung, Akira Meguro, Elham Ashouri, Fei Yu, Nobuhisa Mizuki, Shigeaki Ohno, Raja Rajalingam

**Affiliations:** 1 Ocular Inflammatory Disease Center, Jules Stein Eye Institute, David Geffen School of Medicine at UCLA, University of California Los Angeles, Los Angeles, California, United States of America; 2 Department of Ophthalmology, Yokohama City University School of Medicine, 3–9 Fukuura, Kanazawa-ku, Yokohama, Kanagawa, Japan; 3 UCLA Immunogenetics Center, David Geffen School of Medicine at UCLA, University of California Los Angeles, Los Angeles, California, United States of America; 4 Department of Ophthalmology, Hokkaido University Graduate School of Medicine, N15W7, Kita-ku, Sapporo, Hokkaido, Japan; 5 Immunogenetics and Transplantation Laboratory, Department of Surgery, University of California San Francisco, San Francisco, California, United States of America; Karolinska Institutet, SWEDEN

## Abstract

Cytotoxic T lymphocytes (CTL) and natural killer (NK) cells are killer lymphocytes that provide defense against viral infections and tumor transformation. Analogous to that of CTL, interactions of killer-cell immunoglobulin-like receptors (KIR) with specific human leukocyte antigen (HLA) class I ligands calibrate NK cell education and response. Gene families encoding KIRs and HLA ligands are located on different chromosomes, and feature variation in the number and type of genes. The independent segregation of *KIR* and *HLA* genes results in variable KIR-HLA interactions in individuals, which may impact disease susceptibility. We tested whether *KIR-HLA* combinations are associated with Vogt-Koyanagi-Harada (VKH) disease, a bilateral granulomatous panuveitis that has strong association with HLA-DR4. We present a case control study of 196 VKH patients and 209 controls from a highly homogeneous native population of Japan. *KIR* and *HLA* class I genes were typed using oligonucleotide hybridization method and analyzed using two-tailed Fisher’s exact probabilities. The incidence of *Bx*-*KIR* genotypes was decreased in VKH patients (odds ratio [OR] 0.58, P = 0.007), due primarily to a decrease in centromeric *B-KIR* motif and its associated *KIRs 2DS2*, *2DL2*, *2DS3*, and *2DL5B*. HLA-B22, implicated in poor immune response, was increased in VKH (OR = 4.25, P = 0.0001). HLA-Bw4, the ligand for KIR3DL1, was decreased in VKH (OR = 0.59, P = 0.01). The KIR-HLA combinations 2DL2+C1/C2 and 3DL1+Bw4, which function in NK education, were also decreased in VKH (OR = 0.49, P = 0.012; OR = 0.59, P = 0.013). Genotypes missing these two inhibitory *KIR-HLA* combinations in addition to missing activating *KIRs 2DS2* and *2DS3* were more common in VKH (OR = 1.90, P = 0.002). These results suggest that synergistic hyporesponsiveness of NK cells (due to poor NK education along with missing of activating KIR*s*) and CTL (due to HLA-B22 restriction) fail to mount an effective immune response against viral-infection that may trigger VKH pathogenesis in genetically susceptible individuals, such as HLA-DR4 carriers.

## Introduction

Vogt-Koyanagi-Harada (VKH) disease is characterized by a bilateral granulomatous panuveitis. Extra-ocular symptoms can include aseptic meningitis, poliosis, vitiligo, and sensorineural hearing loss [1]. Clinical and experimental evidence indicates that VKH is an autoimmune disease against melanocytes [2]. Epidemiological studies, including a multistage genome-wide association study of VKH disease, have identified several genetic susceptibility factors that are associated with autoimmune disease [3,4]. Specific human leukocyte antigens (HLA), particularly HLA-DR4, is the strongest genetic risk factor associated with VKH disease [5]. However, the strength of the DR4 association varies among ethnic groups, indicating additional genetic risk factors for VKH.

HLA molecules display a remarkable degree of polymorphism and play a central role in antigen presentation to T lymphocytes that bear αβ T cell receptors [6–8]. The HLA molecules are of two classes that differ in their structure and function. HLA class II molecules (DR, DQ, DP) present antigens to CD4+ T lymphocytes, while HLA class I molecules (HLA-A, -B, -C) present antigens to cytotoxic T lymphocytes (CTL). Certain HLA class I variants also function as ligands for natural killer (NK) cells, the innate lymphocytes that are capable of lysing virally-infected cells in an antigen independent manner quickly without a “priming” period as required for T cells [9] and thus NK cells are crucial for the early control of infections [10]. NK cells discriminate infected cells from healthy cells by measuring the net input of activating and inhibitory signals perceived from infected/transformed cells through a variety of germline-encoded NK cell receptors [11].

Killer cell immunoglobulin-like receptors (KIR) are the key receptors for human NK cells that bind specific HLA class I ligands [12,13]. KIR-mediated NK cell interaction with self-HLA class I molecules begets NK cell maturation (termed licensing or education) and the subsequent ability to survey, recognize, and kill stressed target cells that have lost HLA class I molecules as a consequence of viral infection or tumor transformation [14,15]. The *KIR* gene family displays a high degree of diversity determined not only by the variability in *KIR* gene content between haplotypes, but also by allelic polymorphism [16]. Only four *KIR* genes (*3DL3*, *3DP1*, *2DL4*, and *3DL2*) are present on all haplotypes; these are referred to as `framework' genes. *KIR3DL3* and *3DL2* mark the centromeric and telomeric boundaries of the *KIR* gene complex respectively, while *3DP1* and *2DL4* are located in the middle of the *KIR* gene complex. The 14 kb DNA sequence enriched with L1 repeats between *3DP1* and *2DL4* divides the *KIR* gene complex into two halves: *3DL3* at the 5′-end and *3DP1* at the 3′-end mark the centromeric half, while *2DL4* at the 5′-end and *3DL2* at the 3′-end mark the telomeric half [17]. *KIR2DL1*, *2DL2*, *2DL3* and *2DS2* are only found on the centromeric half of the *KIR* gene complex while *KIR3DL1*, *3DS1*, *2DS1* and *2DS4* are only found on telomeric half. Three *KIR* genes, *2DL5*, *2DS3* and *2DS5*, are found in both centromeric and telomeric locations.

On the basis of gene content, *KIR* haplotypes are broadly classified into two groups, *A* and *B* [18] Group *A* haplotypes have a fixed gene content (*KIR3DL3-2DL3-2DP1-2DL1-3DP1-2DL4-3DL1-2DS4-3DL2*) that encodes four inhibitory KIRs 2DL1, 2DL3, 3DL1 and 3DL2, specific for four major HLA class I ligands, C2, C1, Bw4, and A3/A11 respectively, and an activating KIR 2DS4. In contrast, group *B* haplotypes vary both in number and combination of *KIR* genes, and comprise several genes (*2DL2*, *2DL5*, *2DS1*, *2DS2*, *2DS3*, *2DS5*, *3DS1*) that are not part of the *A* haplotype.

Functional studies and clinical correlations point to HLA-C as the dominant ligands for KIR. All known allotypes of HLA-C have either Asparagine (HLA-Cw1, Cw3, Cw7, Cw8, Cw12, Cw14, and Cw16—termed C1 epitope) or Lysine (HLA-Cw2, Cw4, Cw5, Cw6, Cw15, Cw17 and Cw18—termed C2 epitope) at position 80, located in the F-pocket of the peptide binding groove, and these dimorphic epitopes are recognized by different isoforms of KIR2D [19–22]. Epitopes C1 and C2 are recognized by the inhibitory KIR 2DL2/3 and 2DL1 receptors, respectively. Two unusual HLA-B allotypes (HLA-B*46:*01* and HLA-B*73:*01*) that lack Bw4 and Bw6 epitopes but carry the C1 epitope, are recognized by the inhibitory receptor 2DL2/3 [23]. KIR3DL1 binds to the Bw4 epitope, defined by amino acid residues 77–83 in the α1 domain [24,25], which is present on approximately 40% of the HLA-B allotypes (B13, B27, B37, B38, B44, B47, B49, B51, B52, B53, B57, B58, B59, B63, B77) and 17% of HLA-A allotypes (HLA-A23, 24, 25 and 32). The HLA-A3 and HLA-A11 allotypes carry the A3/11 epitope recognized by KIR3DL2; however, the precise specificity of this receptor has not been defined [26,27]. Peptides bound by HLA-A3/11 influence its binding to KIR3DL2 [28], and HLA-A3/11-KIR3DL2 recognition does not appear to educate NK cells [29]. Very little is known about the ligands for the activating KIRs. Presumably the activating receptors recognize either `induced-self′ (such as MICA and MICB), `altered-self′ (HLA class I molecule loaded with viral peptide) or `non-self′ (pathogen-encoded molecules).

Given that *KIR* genes at chromosome-19 and *HLA* genes at chromosome-6 are polymorphic and display significant variations, the independent segregation of these unlinked gene families produces extraordinary diversity in the number and type of *KIR-HLA* pairs inherited in individuals [13,30]. *KIR-HLA* variation affects the KIR repertoire of NK cell clones, NK cell maturation, the capability to deliver signals, and consequently the NK cell response to human diseases [31]. Previous studies have suggested that activating *KIR* genes and *Bx KIR* haplotypes confer risk for VKH [32–34]. However, these studies are limited by small sample sizes and use of published controls. In order to better define the role of *KIR* and *HLA* variations in VKH, we analyzed a large cohort of VKH patients and healthy controls from a highly homogeneous population of Japan, where the VKH is the second most common cause of uveitis, accounting for 7.0% of cases [35].

## Materials and Methods

### Study subjects

Genome-wide single nucleotide polymorphism (SNP) analysis has clearly shown that most Japanese individuals fall into two main clusters: the Hondo cluster includes most of the individuals from the main islands in Japan, and the Ryukyu cluster includes most of the individuals from Okinawa [36]. The SNPs with the greatest frequency differences between the Hondo and Ryukyu clusters were found in the HLA region in chromosome 6. Moreover, HLA genotyping analysis of 2,005 individuals from 10 regions of Japan found a significant differentiation between Okinawa Island and main island Japanese [37]. Because population stratification can cause spurious associations in case-control studies, we studied the Japanese main islands other than Okinawa Island. One hundred and ninety-six patients diagnosed with VKH disease using published criteria [38] with an average age at onset of 47.9 years (59.7% female) and 209 age-, sex-, ethnically-matched healthy controls with an average age of 44.3 years (59.8% female) were recruited from the main islanders of Yokohama City University (Yokohama City, Japan) and Hokkaido University (Hokkaido, Japan). The control subjects were not related to each other or to the VKH patients in this study. The study was reviewed and approved by the ethics committees at Yokohama City University and Hokkaido University Hospital. All DNA samples received at UCLA were de-identified and only marked as having been obtained from patients with VKH disease or controls. Data obtained were Health Insurance Portability and Accountability Act (HIPAA) compliant, and the study adhered to the tenets of the Declaration of Helsinki.

### *KIR* and *HLA* genotyping

DNA was isolated from peripheral blood using QIAamp blood kit (Quiagen, Valencia, CA). The quality and quantity of DNA was determined by ultraviolet spectrophotometry, and the concentration was adjusted to 100 ng/μl. The presence and absence of 15 *KIR* genes (*2DL1*, *2DL2*, *2DL3*, *2DL4*, *2DL5A*, *2DL5B*, *3DL1*, *3DL2*, *3DL3*, *3DS1*, *2DS1*, *2DS2*, *2DS3*, *2DS4*, and *2DS5*) and two pseudogenes (*KIR2DP1* and *3DP1*) was determined using the Luminex® technology based *KIR* sequence-specific oligonucleotide (SSO) hybridization method (One Lambda, Canoga Park, USA). Briefly, target DNA was PCR-amplified using three separate group-specific primer sets targeting Exons 3+4, 5, and 7–9. Each PCR product was biotinylated, allowing later detection using R-Phycoerythrin-conjugated Strepavidin (SAPE). Each PCR product was denatured and allowed to hybridize to complementary DNA probes conjugated to fluorescently coded microspheres. After washing the beads, bound amplified DNA from the test sample was tagged with SAPE. A flow analyzer, the LABScan™ 100, identified the fluorescent intensity of PE (phycoerythrin) on each microsphere. The assignment of genotypes was based on the reaction pattern compared to patterns associated with published KIR gene sequences in Immuno-Polymorphism Database (http://www.ebi.ac.uk/ipd/kir/). The unique and unusual *KIR* genotypes were further confirmed by retyping using sequence-specific priming-based-polymerase chain reaction typing system (SSP-PCR) and duplex SSP-PCR typing methods [39,40]. UCLA International KIR exchange reference DNA samples, which that covers 20 common *KIR* genotypes, were included as controls for *KIR* genotyping assays. *HLA-A*, *-B*, and *-C* typing was performed by Luminex® technology based SSO hybridization methods (One Lambda, Canoga Park, CA). The KIR-binding HLA class I epitopes were predicted from the *HLA* typing results.

## Data Analysis and Statistical Methods

Differences between controls and patients in the distribution of *KIR* genotypes, HLA allotypes, KIR-binding HLA motifs, and *KIR-HLA* gene combinations were tested by two-tailed Fisher’s exact probabilities (p), with P<0.05 considered to be statistically significant. Since each subject was tested for several *HLA* alleles and the same data were used to compare the frequency of all the detected alleles, it is probable that one of these alleles will by chance deviate significantly. To overcome this error, P was corrected (Pc) by the use of the Bonferroni inequality method (i.e., multiplication of the P values with the number of alleles compared). Odds ratios (OR) with 95% confidence intervals (95% CI) were calculated as the estimate of magnitude of associations of genotypes between patient and control groups. The haplotype frequencies were calculated from the genotype data by the maximum likelihood method using two different computer packages: Arlequin v3.5.2.2 [41] and LinkDos [42], and both analyses provided identical results.

## Results

### Centromeric B haplotype-specific *KIR* genes that are implicated in a stronger NK cell response were decreased in VKH patients

**Fig 1:**
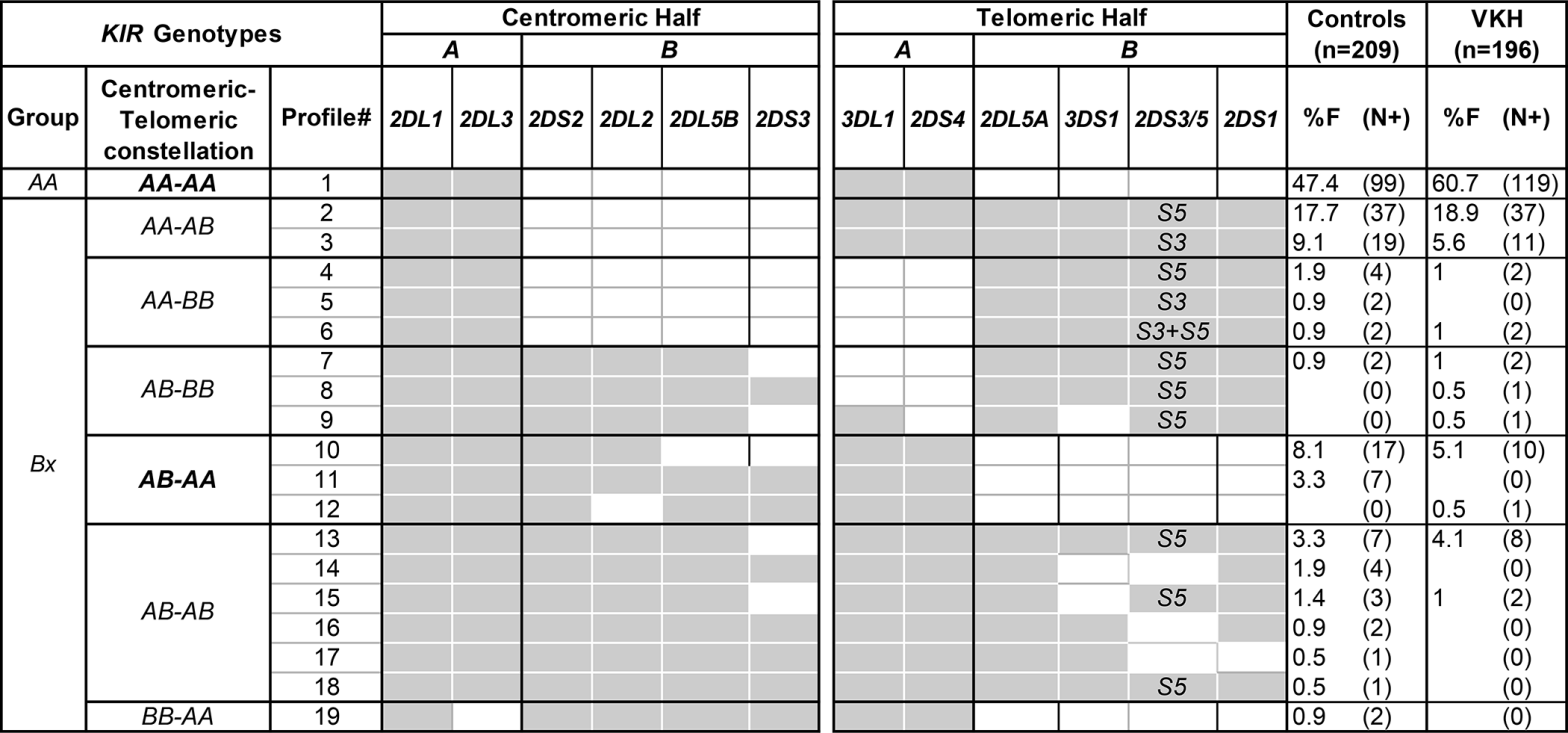
Frequency of *KIR* genotype profiles in patients with Vogt-Koyanagi-Harada (VKH) disease and healthy controls. Nineteen *KIR* genotypes were observed that differ from each other by the presence (indicated by grey shading) of 12 variable *KIR* genes. Genotypes for the centromeric and telomeric parts of the *KIR* locus were assigned according to the presence or absence of *A* and *B* haplotype defining *KIR* genes. Frame work genes (*3DL3*, *3DP1*, *2DL4*, *3DL2*) and pseudogene (*2DP1*) that were observed in all 405 studied subjects are not shown. The frequency of each genotype is presented in percentage frequency (%F) and defined as the number of individuals carrying the genotype (N) divided by the number of individuals studied (n) in the given study group. Significant difference between controls and patients was observed only in the distribution of genotypes with *AA-AA* (p = 0.007) or *AB-AA* (p = 0.05) constellations.

*KIR* gene content profiles were compared between 196 patients with VKH and 209 healthy controls from a homogeneous population of Yokohama and Hokkaido, Japan (Fig 1). A total of 19 genotypes that differ by *KIR* gene content were identified. A significant difference between patients with VKH disease and controls was observed in the distribution of genotypes with the *AA-AA* (P = 0.007) or *AB-AA* (P = 0.05) constellations. The frequency of *Bx KIR* genotypes was decreased in VKH patients compared to controls (OR = 0.58 [95% CI 0.39–0.86], P = 0.007) (Fig 1 and Table 1), and consequently the frequency of *AA KIR* genotypes was increased in VKH patients (OR = 1.72 [95% CI 1.16–2.55], P = 0.007). The decreased frequency of *Bx* genotypes in patients was driven by a decrease in centromeric *Bx* motif (OR = 0.52 [95% CI 0.30–0.88], P = 0.018). Consequently, all variable *B* haplotype-specific *KIR* genes at the centromeric half (*2DL2*, *2DS2*, *2DS3*, *2DL5B*) were reduced in patients (Table 1). There was no difference in the frequencies of telomeric *AA* or telomeric *Bx* groups between patients and controls.

**Table 1:** Frequency of KIR genotypes and KIR genes in patients with VKH disease and healthy controls.

Location	*KIR*	Control (n = 209)		VKH (n = 196)		p-value	OR (95% CI)
		%F	(N+)	%F	(N+)		
	*AA KIR* genotype	47.4	(99)	60.7	(119)	0.007	1.72 (1.16–2.55)
	*Bx KIR* genotype	52.6	(110)	39.3	(77)	0.007	0.58 (0.39–0.86)
	Centromeric *AA*	78.0	(163)	87.2	(171)	0.018	1.93 (1.13–3.29)
	Centromeric *Bx*	22.0	(46)	12.8	(25)	0.018	0.52 (0.30–0.88)
	Telomeric *AA*	59.8	(125)	66.3	(130)		
	Telomeric *Bx*	40.2	(84)	33.7	(66)		
Cen-A	*2DL1*	100	(209)	100	(196)		
Cen-A	*2DL3*	99.0	(207)	100	(196)		
Cen-B	*2DL2*	22.0	(46)	12.2	(24)	0.012	0.49 (0.29–0.85)
Cen-B	*2DS2*	22.0	(46)	12.8	(25)	0.018	0.52 (0.30–0.88)
Cen-B	*2DS3*	19.1	(40)	7.7	(15)	0.0008	0.35 (0.19–0.66)
Cen-B	*2DL5B*	13.9	(29)	7.6	(15)		
Tel-B	*2DL5A*	40.2	(84)	33.7	(66)		
Tel-B	*3DS1*	36.8	(77)	32.1	(63)		
Tel-B	*2DS1*	39.7	(83)	33.7	(66)		
Tel-B	*2DS5*	26.8	(56)	28.1	(55)		
Tel-A	*3DL1*	95.2	(199)	96.4	(189)		
Tel-A	*2DS4*	95.2	(199)	95.9	(188)		

Frequency (%F) of *KIR* gene/genotypes is expressed as percentage and defined as the number of individuals with gene/genotypes (N+) divided by the number of individuals studies in the given study group (n). *KIR3DL3*, *3DP1*, *2DP1*, *2DL4*, and *3DL2* were observed in all 405 subjects. OR: odds ratio, CI: confidence interval, Cen: Centromeric, Tel: Telomeric.

*IR3DL3*, *3DP1*, *2DP1*, *2DL4* and *3DL2* were observed in all 405 subjects.

### Nearly half of the patients with VKH disease carry HLA-B22, which has been implicated in immunological nonresponsiveness

Distributions of HLA-A, -B, and -C allotypes in patients with VKH disease and controls are compared in Table 2. The number of observed HLA-A, B, and -C alleles and the heterozygosities for patients with VKH disease and controls were similar, and did not deviate from Hardy-Weinberg equilibrium (S1 Table). HLA-B54, B56 and Cw1 had a positive association with VKH disease, while HLA-A33, B7, B44, B46, B52 and Cw12 exhibited a negative association with the disease. Of these, the HLA-B54 antigen showed the strongest association, with an odds ratio of 5.39, P = 0.0001 (Pc< 0.005). HLA-B54, B55, and B56 are a group of related antigens originally defined using serologic methods as HLA-B22. The HLA-B22 family members differ by only 1–3 amino acids and display similar peptide binding specificities [43]. Comparison of the entire HLA-B22 group between patients and controls revealed a positive association with VKH disease; HLA-B22 family members were found in 49.5% of patients vs. 17.7% of controls (OR = 4.25 [95% CI 2.70–6.70], P = 0.0001, Pc = 0.002). The association with Cw1 is likely due to its strong linkage disequilibrium with B54 allele, which is evident from all B54 haplotypes carry Cw1 (S2 Table).

**Table 2:** Frequency of HLA-A, -B and -C allotypes in patients with VKH disease and healthy controls.

KIR ligand	HLA Allotypes	Controls (n = 209)		VKH (n = 196)		P-value	OR (95% CI)
		%	(N+)	%	(N+)		
A3/11	A3	1.0	(2)	0.5	(1)		
A3/11	A11	18.2	(38)	24.5	(48)		
Aw4	A24	56.0	(117)	58.7	(115)		
Not a ligand	A1	1.9	(4)	1.5	(3)		
Not a ligand	A2	46.4	(97)	39.8	(78)		
Not a ligand	A26	21.5	(45)	25.0	(49)		
Not a ligand	A30	0.5	(1)		(0)		
Not a ligand	A31	13.9	(29)	18.4	(36)		
Not a ligand	A33	18.2	(38)	8.2	(16)	0.0033	0.40 (0.22–0.74)
Bw4-I80	B38		(0)	0.5	(1)		
Bw4-I80	B51	19.1	(40)	17.3	(34)		
Bw4-I80	B52	21.1	(44)	8.7	(17)	0.0005	0.36 (0.20–0.65)
Bw4-I80	B58	0.5	(1)	0.5	(1)		
Bw4-I80	B59	4.3	(9)	8.2	(16)		
Bw4-T80	B13	1.9	(4)	3.1	(6)		
Bw4-T80	B37	1.4	(3)	1.5	(3)		
Bw4-T80	B44	17.7	(37)	7.1	(14)	0.0015	0.36 (0.19–0.68)
Not a ligand	B7	12.9	(27)	6.1	(12)	0.0275	0.44 (0.22–0.90)
Not a ligand	B35	12.0	(25)	18.4	(36)		
Not a ligand	B39	8.1	(17)	7.7	(15)		
Not a ligand	B48	6.2	(13)	4.1	(8)		
Not a ligand	B60	8.6	(18)	13.8	(27)		
Not a ligand	B61	22.5	(47)	22.4	(44)		
Not a ligand	B54	8.6	(18)	33.7	(66)	0.0001	5.39 (3.06–9.50)
Not a ligand	B55	8.1	(17)	8.7	(17)		
Not a ligand	B56	1.0	(2)	7.1	(14)	0.0015	7.96 (1.79–35.50)
Not a ligand	B62	15.8	(33)	12.2	(24)		
Not a ligand	B67	1.9	(4)	2.6	(5)		
Not a ligand	B71	2.4	(5)	2.0	(4)		
Not a ligand	B75	2.9	(6)	2.6	(5)		
C1	B46	14.8	(31)	4.6	(9)	0.0007	0.28 (0.13–0.60)
C1	Cw1	34.0	(71)	48.0	(94)	0.0046	1.79 (1.20–2.67)
C1	Cw7	26.3	(55)	18.9	(37)		
C1	Cw8	18.2	(38)	15.8	(31)		
C1	Cw9	22.0	(46)	25.5	(50)		
C1	Cw10	19.6	(41)	26.5	(52)		
C1	Cw12	21.1	(44)	9.2	(18)	0.0009	0.38 (0.21–0.68)
C1	Cw14	26.8	(56)	20.9	(41)		
C2	Cw4	8.6	(18)	13.8	(27)		
C2	Cw5	1.0	(2)		(0)		
C2	Cw6	1.9	(4)	2.0	(4)		
C2	Cw15	4.8	(10)	6.1	(12)		

Frequency (%F) of HLA-A, B and C allotypes is expressed as percentage and defined as the number of individuals with gene/genotypes (N+) divided by the number of individuals studies in the given study group (n). OR: odds ratio, CI: confidence interval.

### Frequencies of Bw4, a ligand for KIR3DL1, containing HLA-B allotypes were decreased in patients with VKH disease

Four of the six HLA class I molecules that displayed negative association with VKH disease function as ligands for KIR receptors–HLA-B44 and B52 carry the Bw4 ligand that binds to KIR3DL1 while HLA-B46 and Cw12 carry the C1 ligand that binds to KIR2DL2 and 2DL3. Consequently, the frequency of Bw4 ligand was decreased in patients with VKH disease (OR = 0.59 [95% CI 0.40–0.87], P = 0.010) (Table 3). Bw4 splits into two subsets that differ by the amino acid residue 80, which can be either threonine (Bw4^T80^) or isoleucine (Bw4^I80^). While the frequencies of both subsets were decreased in patients compared to controls, the decrease was statistically significant only for Bw4^T80^ (OR = 0.51 [95% CI 0.30–0.89], P = 0.022). No significant differences were observed in the frequencies of A3/11, Aw4 (Bw4 ligand on HLA-A23, 24, 25 and 32), C1 and C2 ligands between controls and patients (Table 3). However, genotypes with ≥3 copies of C1-ligands were reduced in patients (OR = 0.24 [95% CI 0.11–0.55], P = 0.0003).

**Table 3:** Frequency of KIR-binding HLA class I ligands in patients with VKH disease and healthy controls.

Genotypes	Controls (n = 209)		VKH (n = 196)		p-value	OR (95% CI)
	%F	(N+)	%F	(N+)		
HLA-A3/11	19.1	(40)	25.0	(49)		
A3	0.9	(2)	0.5	(1)		
A11	18.2	(38)	24.5	(48)		
A3+A11		(0)		(0)		
HLA-Bw4	55.5	(116)	42.4	(83)	0.0097	0.59 (0.40–0.87)
Bw4^T80^	20.6	(43)	11.7	(23)	0.0216	0.51 (0.30–0.89)
Bw4^I80^	41.6	(87)	33.7	(66)		
HLA-Aw4	56.0	(117)	58.7	(115)		
Bw4/Aw4	75.1	(157)	71.4	(140)		
*> 2 copies* (Bw4+Bw4/ Bw4+Aw4/ Aw4+Aw4)	37.3	(78)	29.6	(58)		
HLA-C1	99.0	(207)	98.5	(193)		
HLA-C2	16.3	(34)	21.4	(42)		
C1+C1	83.7	(175)	78.5	(154)		
C2+C2	0.9	(2)	1.5	(3)		
C1+C2	15.3	(32)	19.9	(39)		
> 3 copies of C1	14.8	(31)	4.1	(8)	0.0003	0.24 (0.11–0.55)

Frequency (%F) of HLA class I ligand is expressed as percentage and defined as the number of individuals with the ligand (N+) divided by the number of individuals studies in the given study group (n). OR: odds ratio, CI: confidence interval.

The distributions of the 30 most frequent *HLA* haplotypes were compared between patients with VKH disease and controls, as well as published data in mainland Japan and Okinawa, Japan (S2 Table) [37,44,45]. The frequencies of the *HLA* haplotypes in our control group are similar to published data from mainland Japan, but differ significantly from data from Okinawa, Japan. Three haplotypes (A24-B54-Cw1, A24-B60-Cw10, A11-B54-Cw1) were positively associated with VKH (OR = 5.06 [95% CI 2.52–10.14], P = 0.0001; OR = 5.28 [95% CI 1.49–18.67], P = 0.006; and OR = 7.35 [95% CI 1.64–33.02], P = 0.003, respectively) (S2 Table). Three other haplotypes (A24-B52-Cw12, A33-B44-Cw14, A2-B46-Cw1) were negatively associated with VKH (OR = 0.36 [95% CI 0.20–0.67], P = 0.002; OR = 0.34 [95% CI 0.17–0.67], P = 0.002; and OR = 0.22 [95% CI 0.09–0.52], P = 0.001, respectively). All these six haplotypes possess at least 2 KIR ligands (A11, A24, B44, B46, B52, Cw1, Cw10, Cw12, Cw14) including at least one C1 ligand (Cw1, Cw10, Cw12, Cw14, B46), and haplotype A2-B46-Cw1 possess 2 copies of C1 ligands (B46 and Cw1). HLA-B54 appears in two out of the three haplotypes associated with VKH disease.

### Inhibitory KIR-HLA combinations that calibrate NK education and response to infection are decreased in patients with VKH disease

**Fig 2:**
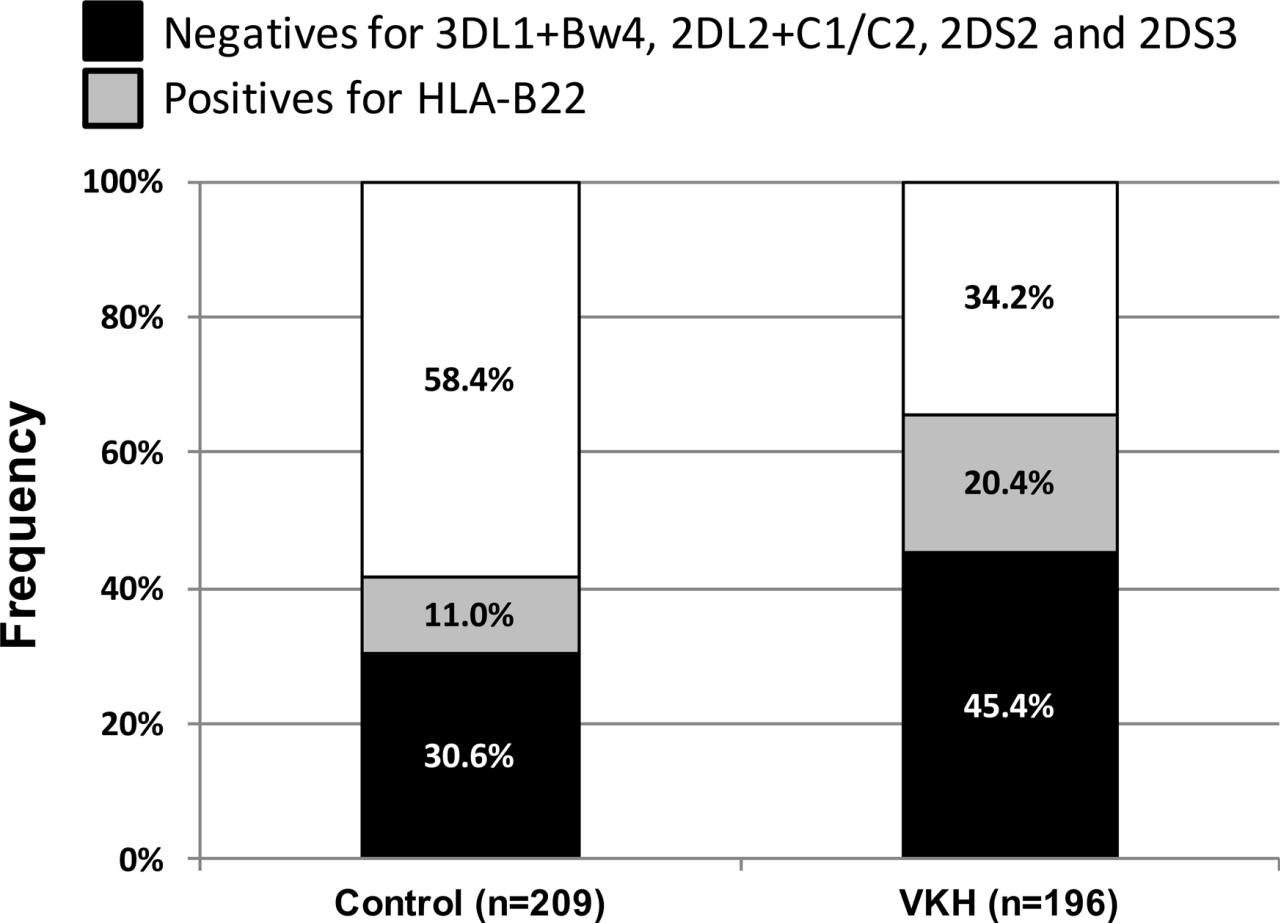
*KIR-HLA* genotypes predictive of reduced NK cell education and response are associated with VKH. 45.4% of VKH group lack the following 4 KIR/HLA factors: 3DL1+Bw4, 2DL2+C1/C2, 2DS2 and 2DS3, while only 30.6% of the controls display this genotypes (p = 0.002, OR = 1.9 [95% CI 1.28–2.83]).

2DL2/3+C1, 2DL1+C2 and 3DL1+Bw4 are well-defined inhibitory KIR-HLA pairs that play a pivotal role in NK cell education and maturation. The decreases in 2DL2 and Bw4 discussed above were associated with concomitant reductions in 2DL2+C1/C2 and 3DL1+Bw4 combinations in patients with VKH disease (OR = 0.49 [95% CI 0.29–0.85], P = 0.012; OR = 0.59 [95% CI 0.40–0.88], P = 0.013 respectively) (Table 4). The difference in the frequency of 3DL1+Bw4 between groups was driven by Bw4^T80^ (OR = 0.50 [95% CI 0.29–0.88], P = 0.02). The VKH population demonstrates a higher incidence of genotypes that lack these two KIR-HLA pairs, as well as two activating KIRs (2DS2 and 2DS3), compared to controls (45.4% vs. 30.6%, OR 1.90 [95% CI 0.28–2.83], P = 0.002) (Fig 2). Within the remaining 54.6% of patients with VKH disease, 20.4% carry HLA-B22, and thus 65.8% of patients either lack 4 KIR/HLA factors (3DL1+Bw4, 2DL2+C1/C2, 2DS2 and 2DS3) or carry HLA-B22, while only 41.6% of controls display such genotypes.

**Table 4:** Frequency of KIR-HLA class I ligand combination in patients with VKH disease and healthy controls.

KIR-HLA combinations	Controls (n = 209)		VKH (n = 196)		p-value	OR (95% CI)
	%F	(N+)	%F	(N+)		
2DL1+C2	16.3	(34)	21.4	(42)		
2DL2+C1/2	22.0	(46)	12.2	(24)	0.012	0.49 (0.29–0.85)
2DL3+C1	98.1	(205)	98.5	(193)		
2DL2/3+C1	99.0	(207)	98.5	(193)		
3DL1+Bw4	52.6	(110)	39.8	(78)	0.013	0.59 (0.40–0.88)
3DL1+Bw4^T80^	20.1	(42)	11.2	(22)	0.02	0.50 (0.29–0.88)
3DL1+Bw4^I80^	39.2	(82)	31.6	(62)		
3DL1+Aw4	54.1	(113)	57.7	(113)		
3DL1+Aw4/Bw4	75.1	(157)	73.5	(144)		
3DL2+A3/11	19.1	(40)	25.0	(49)		

Frequency (%F) of KIR-HLA class I ligand combinations expressed as percentage and defined as the number of individuals with the KIR-HLA combination (N+) divided by the number of individuals studies in the given study group (n). OR: odds ratio, CI: confidence interval.

## Discussion

Among the genetic factors that confer risk for autoimmune disease are the *HLA* and *KIR* genes, which are highly polymorphic and are very important regulators of the innate and adaptive immune response in humans. In this study, we found that VKH is associated with a decrease in the centromeric *Bx* motif and its associated *KIR* genes– *2DL2*, *2DS2*, *2DS3*, and *2DL5B*. This is in contrast to the previous findings in Mestizo [32], Saudi Arabian [34], and Japanese [33] populations, that were found to have an increased frequency of *KIR B*-haplotypes and certain activating *KIR* genes. This inconsistency is likely attributed to small sample size (<30 patients) and use of published controls in previous studies. The present study examined a much larger cohort of 196 patients with VKH disease and 209 healthy controls from a homogenous native Japanese population of Yokohama and Hokkaido, Japan. The *HLA* haplotype frequency in the control population is comparable to published data from mainland Japan, indicating the cases and controls in this study were of Japanese ancestry and were well matched.

The centromeric-*B KIR* gene complex may decrease risk for VKH disease by bolstering the immune response against infectious and other environmental triggers for autoimmunity. The clearest demonstration of the clinical benefit of specific *KIR* genotypes derives from analyses in *HLA*-matched hematopoietic stem cell transplantation (HSCT), which revealed the beneficial effect of having a centromeric-*B KIR* motif in the donor [46–49]. These studies indicate that NK cells generated from centromeric-*B KIR* motif carriers are strongly reactive and may; 1) protect against graft versus host disease (GvHD) by depleting host antigen presenting cells; 2) facilitate engraftment by elimination of host immune barriers; 3) kill residual host tumor to protect against relapse, and; 4) decrease infectious complications. The clinical benefit conferred by the centromeric-*B KIR* motif could result from the presence of activating *KIRs 2DS2* and *2DS3*. These two activating KIRs have no detectable avidity for HLA class I [50,51], but may recognize different ligands associated with infection or tumor transformation. Many association studies have demonstrated that activating *KIR* genes protect against infectious disease [52]. Therefore, carrying activating *KIRs 2DS2* and *2DS3* could represent a better protection against viruses, microorganisms, or other environmental precipitants of VKH disease. A similar protective role for *B KIR* genotypes and activating *KIRs* was recognized in other autoimmune diseases that were linked to viral infections, such as Multiple Sclerosis [53] and Pemphigus Foliaceus [54]. It is also important to consider the possibility of other polymorphic *KIR* genes that are located at the centromeric-*B* motif, such as *3DL3* and *2DL5B* for which ligands and functions are unknown could also confer decreased risk for developing VKH disease.

*HLA-B* is the most polymorphic gene in humans, encoding over 3,130 distinct protein variants in human populations [55]. A striking finding of the current study is the strong association of HLA-B54 antigen (and related antigen group B22) with VKH disease. Interestingly, the association of HLA-B22 (originally called HLA-BW22J) and VKH in Japanese was first reported almost 40 years ago [56,57]. However, it could not be replicated in a subsequent study with 9 VKH patients from California [58]. The discrepancy between these two studies might be due to a different ethnic background, small sample size, and/or the serological HLA typing method used that was known to have up to 25% discrepancy compared to DNA-based methods [59]. Furthermore, these first studies were soon overshadowed by the strong association of HLA-DR4 in Japanese patients [60]. It is important to note that the *HLA-DR4* is in strong linkage disequilibria with *HLA-B54* and *HLA-Cw1*, one of the common haplotypes in a Japanese population, but uncommon in other parts of the world [61].

Although we did not test the current cohorts for HLA-DR types, published association studies in Japanese VKH patients [60,62–64] and the strong linkage disequilibrium between HLA-B and HLA-DR loci prophesies a potential positive association of HLA-DR4 with the present cohort of VKH patients [61]. Ocular infiltrating CD4+ T cells from DR4+ patients with VKH disease were shown to recognize self-peptides from melanocytes (tyrosinase_450-462_ and gp100_44-59_) that share high sequence homology with cytomegalovirus envelope glycoprotein H (CMV-egH_290–302_), suggesting that CMV infection may stimulate the production of T cells that cross-react with tyrosinase by a mechanism of molecular mimicry [65,66]. In addition to VKH, HLA-DR4 and the linked HLA-B54 are associated with other autoimmune diseases in Japanese patients, including Type 1 Diabetes [67] and rheumatoid arthritis [68], suggesting a common underlying mechanism for autoimmune diseases in Japanese population.

HLA-B22 confers susceptibility to several viral infectious diseases. For instance, B22 was associated with rapidly progressive disease in human immunodeficiency virus (HIV) infected individuals [69,70], immunological nonresponse to hepatitis B virus surface antigen [71], myelopathy due to human T-lymphotropic virus type I [72], and progression of liver injury [73] and unresponsiveness to interferon-α treatment in hepatitis C virus infection [74]. Nearly 50% of the VKH patients studied here carry HLA-B22, which implicates a B22-mediated immunological nonresponsiveness to viral infections, which may play a role in the pathogenesis of VKH disease. Consistent with this possibility, B lymphocyte cell lines established from patients with VKH disease contain greater amounts of Epstein-Barr virus (EBV) DNA, and express more EBV viral antigens than those established from patients with other types of uveitis, indicating EBV-specific nonresponsiveness in VKH patients [75].

**Fig 3:**
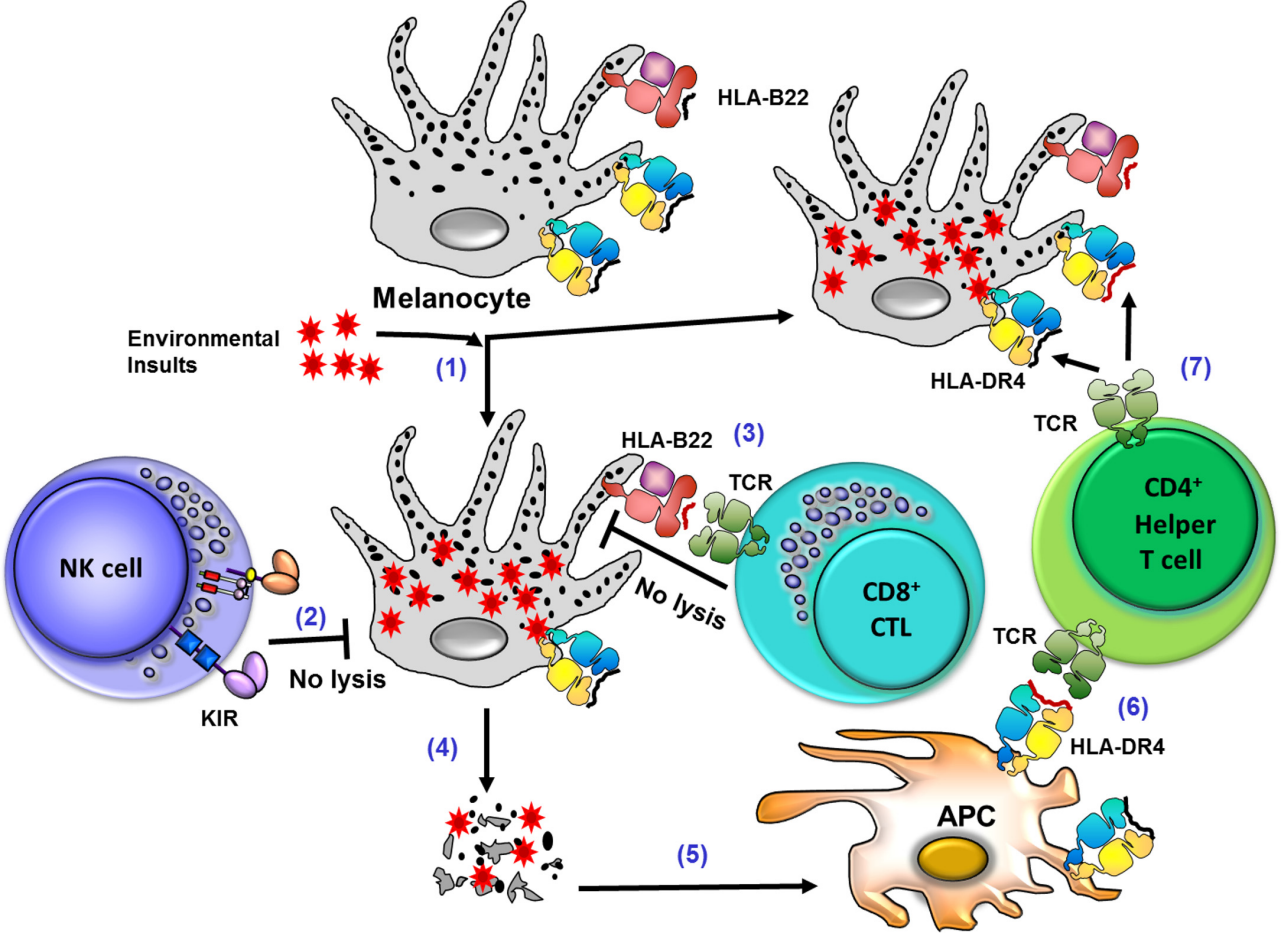
Proposed mechanism for the Immunogenetic basis of VKH pathogenesis. (1) Foreign immunogenic peptides, altered self peptides, or self peptides usually sequestered in melanocytes or other melanin bearing cells, as a result of intracellular infections or cell damage secondary to such processes as melanoma therapy, may trigger VKH in susceptible individuals. For example, CMV-infected melanocytes process and display viral peptides in the context of HLA class I molecules, such as HLA-B22 in susceptible individuals; (2) NK cells of those susceptible individuals that are missing two of the three inhibitory KIR-HLA interactions (that involve in NK cell licensing/education) and in addition to missing activating *KIRs 2DS2* and *2DS3* (that are implicated in a stronger NK cell response) are more likely hyporesponsive against virally-infected melanocytes; (3) CD8+ cytotoxic T cells (CTL) that kill cells that are infected with viruses with toxic mediators can be hyporesponsive if the infected melanocytes present viral peptides in the context of HLA-B22 in susceptible individuals; (4) Independent or synergistic unresponsiveness of NK cells and CTL could be unable to efficiently contain the viral infection, and resulting in the destruction of the infected melanocyte and its membrane; (5) Immune surveillance cells, such as antigen presenting cells (APC) ingest viral antigens and other cellular debris from melanocytes. In individuals whose melanoma cells are destroyed by aggressive and successful therapy may similarly release otherwise sequestered melanin related antigens; (6) APC present the processed CMV envelope glycoprotein H (CMV-egH_290–302_) as well as self-peptides from melanocytes (tyrosinase_450-462_ and gp100_44-59_) to CD4+ helper T cells via HLA-DR4, a class II molecule strongly associated with VKH in Japanese; (7) Because of the high sequence homology between self-peptides from melanocytes (tyrosinase_450-462_ and gp100_44-59_) and CMV envelope glycoprotein H (CMV-egH_290–302_), CD4+ T cells may cross-react with tyrosinase by a mechanism of molecular mimicry and trigger an autoimmune response against pigment expressing melanocytes, precipitating VKH disease. Although the model explains the potential role of observed KIR-HLA association, appropriate functional studies are required to define the mechanism by which CTL and NK cells contribute to/protect against VKH pathogenesis.

The interaction of inhibitory KIR receptors with specific HLA class I molecules mediates NK tolerance to self while conferring functional competence [14,15,76–78]. NK cells generated from *KIR3DL1-Bw4* carriers were shown to strongly influence progression of acquired immune deficiency syndrome (AIDS) and plasma HIV RNA levels [79]. Among the three well-defined inhibitory KIR-HLA combinations that involve in NK cell education and maturation (2DL2/3+C1, 2DL1+C2 and 3DL1+Bw4), two (2DL2+C1/C2 and 3DL1+Bw4) were decreased in patients with VKH disease, suggesting a protective role of these inhibitory KIR-HLA combinations. Presumably the absence of these two inhibitory KIR-HLA interactions results in the generation of mostly unlicensed NK cells that fail to mount a vigorous NK response against infections implicated in VKH pathogenesis. HLA-B22 mediated unresponsiveness could act independently as well as synergistically to modify VKH disease susceptibility. These results suggest that a hyporesponsiveness of NK cells due to poor education plus missing of activating KIRs, as well as by CTLs due to nonresponsive HLA-B22, play an important role in VKH pathogenesis following viral infections in genetically susceptible individuals, such as HLA-DR4 carriers. A comprehensive model that summarizes potential mechanisms for the Immunogenetic basis of VKH pathogenesis is presented in Fig 3.

Immunotherapy, including tumor infiltrating lymphocyte therapy, interleukin-2, and the CTLA inhibitor ipilimumab, promotes T cell activation and survival in order to augment the immune response against neoplastic cells, but inadvertently tips the balance toward autoimmunity including the development of VKH [80–82]. One of the authors (RDL) has seen VKH develop in a patient whose malignant melanoma was successfully treated with BRAF and MEK inhibitors, suggesting that the release of antigens from the melanoma may induce the autoimmune response to intraocular antigens in a genetically predisposed patient (unpublished data). *KIR* and *HLA* genotyping may be useful in determining the risk of developing uveitis patients either receiving immunostimulatory therapy for cancer or MEK/BRAF treatment for malignant melanoma in particular. Patients found to be genetically at risk could then be monitored closely by an ophthalmologist.

A high frequency of HLA-B22 and a low frequency of the *Bx KIR* genotype is the characteristic genetic composition of the Japanese population, which may play a role in pathogenesis and be part of why VKH is more common in Japanese than other populations. It is important to recognize the genetic analysis alone is unlikely to distinguish the contribution of predisposing *KIR-HLA* factors, and functional studies will be required to define the mechanism by which CTL and NK cells contribute to/protect against VKH pathogenesis. The understanding of the mechanisms underlying the genetic basis for VKH susceptibility could have important implications for treatment in the clinic, providing new targets for therapeutic manipulation of disease process.

## Supporting Information

S1 Table Baseline characteristics of HLA genotyping for patients with VKH disease and healthy controls. (XLS)

S2 Table Frequency of the 30 most common HLA-A-B-C haplotypes in patients with VKH disease and healthy controls. (XLS)

## References

[1] Sakata VM, da Silva FT, Hirata CE, de Carvalho JF, Yamamoto JH. Diagnosis and classification of Vogt-Koyanagi-Harada disease. Autoimmun Rev. 2014; 13: 550–555. 10.1016/j.autrev.2014.01.023 24440284

[2] Lavezzo MM, Sakata VM, Morita C, Rodriguez EE, Abdallah SF, da Silva FT, et al Vogt-Koyanagi-Harada disease: review of a rare autoimmune disease targeting antigens of melanocytes. Orphanet J Rare Dis. 2016; 11: 29 10.1186/s13023-016-0412-4 27008848 PMC4806431

[3] Ng JY, Luk FO, Lai TY, Pang CP. Influence of molecular genetics in Vogt-Koyanagi-Harada disease. J Ophthalmic Inflamm Infect. 2014; 4: 20 10.1186/s12348-014-0020-1 25097674 PMC4105881

[4] Hou S, Du L, Lei B, Pang CP, Zhang M, Zhuang W, et al Genome-wide association analysis of Vogt-Koyanagi-Harada syndrome identifies two new susceptibility loci at 1p31.2 and 10q21.3. Nat Genet. 2014; 46: 1007–1011. 10.1038/ng.3061 25108386

[5] Shi T, Lv W, Zhang L, Chen J, Chen H. Association of HLA-DR4/HLA-DRB1*04 with Vogt-Koyanagi-Harada disease: a systematic review and meta-analysis. Sci Rep. 2014; 4: 6887 10.1038/srep06887 25382027 PMC4225552

[6] Klein J, Sato A. The HLA system. First of two parts. N Engl J Med. 2000; 343: 702–709. 10974135 10.1056/NEJM200009073431006

[7] Klein J, Sato A. The HLA system. Second of two parts. N Engl J Med. 2000; 343: 782–786. 10984567 10.1056/NEJM200009143431106

[8] Marsh SG, Albert ED, Bodmer WF, Bontrop RE, Dupont B, Erlich HA, et al Nomenclature for factors of the HLA system, 2010. Tissue Antigens. 2010; 75: 291–455. 10.1111/j.1399-0039.2010.01466.x 20356336 PMC2848993

[9] Trinchieri G. Biology of natural killer cells. Adv Immunol. 1989; 47: 187–376. 2683611 10.1016/S0065-2776(08)60664-1 PMC7131425

[10] Lee SH, Miyagi T, Biron CA. Keeping NK cells in highly regulated antiviral warfare. Trends Immunol. 2007; 28: 252–259. 17466596 10.1016/j.it.2007.04.001

[11] Lanier LL. NK cell recognition. Annu Rev Immunol. 2005; 23: 225–274. 15771571 10.1146/annurev.immunol.23.021704.115526

[12] Parham P. MHC class I molecules and KIRs in human history, health and survival. Nat Rev Immunol. 2005; 5: 201–214. 15719024 10.1038/nri1570

[13] Rajalingam R. Human diversity of killer cell immunoglobulin-like receptors and disease. Korean J Hematol. 2011; 46: 216–228. 10.5045/kjh.2011.46.4.216 22259627 PMC3259513

[14] Kim S, Poursine-Laurent J, Truscott SM, Lybarger L, Song YJ, Yang L, et al Licensing of natural killer cells by host major histocompatibility complex class I molecules. Nature. 2005; 436: 709–713. 16079848 10.1038/nature03847

[15] Anfossi N, Andre P, Guia S, Falk CS, Roetynck S, Stewart CA, et al Human NK cell education by inhibitory receptors for MHC class I. Immunity. 2006; 25: 331–342. 16901727 10.1016/j.immuni.2006.06.013

[16] Wilson MJ, Torkar M, Haude A, Milne S, Jones T, Sheer D, et al Plasticity in the organization and sequences of human KIR/ILT gene families. Proc Natl Acad Sci U S A. 2000; 97: 4778–4783. 10781084 10.1073/pnas.080588597 PMC18309

[17] Rajalingam R. Overview of the killer cell immunoglobulin-like receptor system. Methods Mol Biol. 2012; 882: 391–414. 10.1007/978-1-61779-842-9_23 22665247

[18] Uhrberg M, Valiante NM, Shum BP, Shilling HG, Lienert-Weidenbach K, Corliss B, et al Human diversity in killer cell inhibitory receptor genes. Immunity. 1997; 7: 753–763. 9430221 10.1016/s1074-7613(00)80394-5

[19] Colonna M, Spies T, Strominger JL, Ciccone E, Moretta A, Moretta L, et al Alloantigen recognition by two human natural killer cell clones is associated with HLA-C or a closely linked gene. Proc Natl Acad Sci U S A. 1992; 89: 7983–7985. 1518825 10.1073/pnas.89.17.7983 PMC49839

[20] Colonna M, Borsellino G, Falco M, Ferrara GB, Strominger JL. HLA-C is the inhibitory ligand that determines dominant resistance to lysis by NK1- and NK2-specific natural killer cells. Proc Natl Acad Sci U S A. 1993; 90: 12000–12004. 8265660 10.1073/pnas.90.24.12000 PMC48113

[21] Wagtmann N, Rajagopalan S, Winter CC, Peruzzi M, Long EO. Killer cell inhibitory receptors specific for HLA-C and HLA-B identified by direct binding and by functional transfer. Immunity. 1995; 3: 801–809. 8777725 10.1016/1074-7613(95)90069-1

[22] Winter CC, Long EO. A single amino acid in the p58 killer cell inhibitory receptor controls the ability of natural killer cells to discriminate between the two groups of HLA-C allotypes. J Immunol. 1997; 158: 4026–4028. 9126959

[23] Moesta AK, Norman PJ, Yawata M, Yawata N, Gleimer M, Parham P. Synergistic polymorphism at two positions distal to the ligand-binding site makes KIR2DL2 a stronger receptor for HLA-C than KIR2DL3. J Immunol. 2008; 180: 3969–3979. 18322206 10.4049/jimmunol.180.6.3969

[24] Gumperz JE, Litwin V, Phillips JH, Lanier LL, Parham P. The Bw4 public epitope of HLA-B molecules confers reactivity with natural killer cell clones that express NKB1, a putative HLA receptor. J Exp Med. 1995; 181: 1133–1144. 7532677 10.1084/jem.181.3.1133 PMC2191933

[25] Cella M, Longo A, Ferrara GB, Strominger JL, Colonna M. NK3-specific natural killer cells are selectively inhibited by Bw4-positive HLA alleles with isoleucine 80. J Exp Med. 1994; 180: 1235–1242. 7931060 10.1084/jem.180.4.1235 PMC2191670

[26] Pende D, Biassoni R, Cantoni C, Verdiani S, Falco M, di Donato C, et al The natural killer cell receptor specific for HLA-A allotypes: a novel member of the p58/p70 family of inhibitory receptors that is characterized by three immunoglobulin-like domains and is expressed as a 140-kD disulphide-linked dimer. J Exp Med. 1996; 184: 505–518. 8760804 10.1084/jem.184.2.505 PMC2192700

[27] Dohring C, Scheidegger D, Samaridis J, Cella M, Colonna M. A human killer inhibitory receptor specific for HLA-A1,2. J Immunol. 1996; 156: 3098–3101. 8617928

[28] Hansasuta P, Dong T, Thananchai H, Weekes M, Willberg C, Aldemir H, et al Recognition of HLA-A3 and HLA-A11 by KIR3DL2 is peptide-specific. Eur J Immunol. 2004; 34: 1673–1679. 15162437 10.1002/eji.200425089

[29] Yawata M, Yawata N, Draghi M, Partheniou F, Little AM, Parham P. MHC class I-specific inhibitory receptors and their ligands structure diverse human NK-cell repertoires toward a balance of missing self-response. Blood. 2008; 112: 2369–2380. 10.1182/blood-2008-03-143727 18583565 PMC2532809

[30] Du Z, Gjertson DW, Reed EF, Rajalingam R. Receptor-ligand analyses define minimal killer cell Ig-like receptor (KIR) in humans. Immunogenetics. 2007; 59: 1–15. 17103212 10.1007/s00251-006-0168-4

[31] Rajagopalan S, Long EO. Understanding how combinations of HLA and KIR genes influence disease. J Exp Med. 2005; 201: 1025–1029. 15809348 10.1084/jem.20050499 PMC2213130

[32] Levinson RD, Du Z, Luo L, Holland GN, Rao NA, Reed EF, et al KIR and HLA gene combinations in Vogt-Koyanagi-Harada disease. Hum Immunol. 2008; 69: 349–353. 10.1016/j.humimm.2008.04.005 18571006

[33] Levinson RD, Okada AA, Ashouri E, Keino H, Rajalingam R. Killer cell immunoglobulin-like receptor gene-cluster 3DS1-2DL5-2DS1-2DS5 predisposes susceptibility to Vogt-Koyanagi-Harada syndrome in Japanese individuals. Hum Immunol. 2010; 71: 192–194. 10.1016/j.humimm.2009.11.001 19897003

[34] Sheereen A, Gaafar A, Iqneibi A, Eldali A, Tabbara KF, Adra C, et al A study of KIR genes and HLA-C in Vogt-Koyanagi-Harada disease in Saudi Arabia. Mol Vis. 2011; 17: 3523–3528. 22219647 PMC3250373

[35] Iwahashi C, Okuno K, Hashida N, Nakai K, Ohguro N, Nishida K. Incidence and clinical features of recurrent Vogt-Koyanagi-Harada disease in Japanese individuals. Jpn J Ophthalmol. 2015; 59: 157–163. 10.1007/s10384-015-0377-1 25808016

[36] Yamaguchi-Kabata Y, Nakazono K, Takahashi A, Saito S, Hosono N, Kubo M, et al Japanese population structure, based on SNP genotypes from 7003 individuals compared to other ethnic groups: effects on population-based association studies. Am J Hum Genet. 2008; 83: 445–456. 10.1016/j.ajhg.2008.08.019 18817904 PMC2561928

[37] Nakaoka H, Mitsunaga S, Hosomichi K, Shyh-Yuh L, Sawamoto T, Fujiwara T, et al Detection of ancestry informative HLA alleles confirms the admixed origins of Japanese population. PLoS One. 2013; 8: e60793 10.1371/journal.pone.0060793 23577161 PMC3618337

[38] Read RW, Holland GN, Rao NA, Tabbara KF, Ohno S, Arellanes-Garcia L, et al Revised diagnostic criteria for Vogt-Koyanagi-Harada disease: report of an international committee on nomenclature. Am J Ophthalmol. 2001; 131: 647–652. 11336942 10.1016/s0002-9394(01)00925-4

[39] Ashouri E, Farjadian S, Reed EF, Ghaderi A, Rajalingam R. KIR gene content diversity in four Iranian populations. Immunogenetics. 2009; 61: 483–492. 10.1007/s00251-009-0378-7 19521696 PMC2706385

[40] Rajalingam R, Ashouri E. Gene-specific PCR typing of killer cell immunoglobulin-like receptors. Methods Mol Biol. 2013; 1034: 239–255. 10.1007/978-1-62703-493-7_12 23775740

[41] Excoffier L, Lischer HE. Arlequin suite ver 3.5: a new series of programs to perform population genetics analyses under Linux and Windows. Mol Ecol Resour. 2010; 10: 564–567. 10.1111/j.1755-0998.2010.02847.x 21565059

[42] Garnier-Gere P, Dillmann C. A computer program for testing pairwise linkage disequilibria in subdivided populations. J Hered. 1992; 83: 239. 10.1093/oxfordjournals.jhered.a111204 1624774

[43] Barber LD, Gillece-Castro B, Percival L, Li X, Clayberger C, Parham P. Overlap in the repertoires of peptides bound in vivo by a group of related class I HLA-B allotypes. Curr Biol. 1995; 5: 179–190. 7743181 10.1016/s0960-9822(95)00039-x

[44] Yawata M, Yawata N, McQueen KL, Cheng NW, Guethlein LA, Rajalingam R, et al Predominance of group A KIR haplotypes in Japanese associated with diverse NK cell repertoires of KIR expression. Immunogenetics. 2002; 54: 543–550. 12439616 10.1007/s00251-002-0497-x

[45] Yawata M, Yawata N, Draghi M, Little AM, Partheniou F, Parham P. Roles for HLA and KIR polymorphisms in natural killer cell repertoire selection and modulation of effector function. J Exp Med. 2006; 203: 633–645. 16533882 10.1084/jem.20051884 PMC2118260

[46] Cooley S, Trachtenberg E, Bergemann TL, Saeteurn K, Klein J, Le CT, et al Donors with group B KIR haplotypes improve relapse-free survival after unrelated hematopoietic cell transplantation for acute myelogenous leukemia. Blood. 2009; 113: 726–732. 10.1182/blood-2008-07-171926 18945962 PMC2628378

[47] Cooley S, Weisdorf DJ, Guethlein LA, Klein JP, Wang T, Le CT, et al Donor selection for natural killer cell receptor genes leads to superior survival after unrelated transplantation for acute myelogenous leukemia. Blood. 2010; 116: 2411–2419. 10.1182/blood-2010-05-283051 20581313 PMC2953880

[48] Stringaris K, Adams S, Uribe M, Eniafe R, Wu CO, Savani BN, et al Donor KIR Genes 2DL5A, 2DS1 and 3DS1 are associated with a reduced rate of leukemia relapse after HLA-identical sibling stem cell transplantation for acute myeloid leukemia but not other hematologic malignancies. Biol Blood Marrow Transplant. 2010; 16: 1257–1264. 10.1016/j.bbmt.2010.03.004 20302958 PMC3801172

[49] Hsu KC, Gooley T, Malkki M, Pinto-Agnello C, Dupont B, Bignon JD, et al KIR ligands and prediction of relapse after unrelated donor hematopoietic cell transplantation for hematologic malignancy. Biol Blood Marrow Transplant. 2006; 12: 828–836. 16864053 10.1016/j.bbmt.2006.04.008

[50] Hilton HG, Vago L, Older Aguilar AM, Moesta AK, Graef T, Abi-Rached L, et al Mutation at positively selected positions in the binding site for HLA-C shows that KIR2DL1 is a more refined but less adaptable NK cell receptor than KIR2DL3. J Immunol. 2012; 189: 1418–1430. 10.4049/jimmunol.1100431 22772445 PMC3439511

[51] Saulquin X, Gastinel LN, Vivier E. Crystal structure of the human natural killer cell activating receptor KIR2DS2 (CD158j). J Exp Med. 2003; 197: 933–938. 12668644 10.1084/jem.20021624 PMC2193886

[52] Kulkarni S, Martin MP, Carrington M. The Yin and Yang of HLA and KIR in human disease. Semin Immunol. 2008; 20: 343–352. 10.1016/j.smim.2008.06.003 18635379 PMC3501819

[53] Fusco C, Guerini FR, Nocera G, Ventrella G, Caputo D, Valentino MA, et al KIRs and their HLA ligands in remitting-relapsing multiple sclerosis. J Neuroimmunol. 2010; 229: 232–237. 10.1016/j.jneuroim.2010.08.004 20826009

[54] Augusto DG, Lobo-Alves SC, Melo MF, Pereira NF, Petzl-Erler ML. Activating KIR and HLA Bw4 ligands are associated to decreased susceptibility to pemphigus foliaceus, an autoimmune blistering skin disease. PLoS One. 2012; 7: e39991 10.1371/journal.pone.0039991 22768326 PMC3388041

[55] Robinson J, Halliwell JA, Hayhurst JD, Flicek P, Parham P, Marsh SGE. The IPD and IMGT/HLA database: allele variant databases. Nucleic Acids Research. 2015; 43: D423–D431. 10.1093/nar/gku1161 25414341 PMC4383959

[56] Tagawa Y, Sugiura S, Yakura H, Wakisaka A, Aizawa M. Letter: HLA and Vogt-Koyanagh-Harada syndrome. N Engl J Med. 1976; 295: 173. 10.1056/NEJM197607152950322 1272341

[57] Yakura H, Wakisaka A, Aizawa M, Itakura K, Tagawa Y. HLA-D antigen of Japanese origin (LD-Wa) and its association with Vogt-Koyanagi-Harada syndrome. Tissue Antigens. 1976; 8: 35–42. 60795 10.1111/j.1399-0039.1976.tb00549.x PMC8367706

[58] Douglass SN, Douglass JM. Letter: Hla and vogt-koyanagi-harada syndrome. N Engl J Med. 1976; 295: 788. 10.1056/NEJM197609302951417 958269

[59] Opelz G, Mytilineos J, Scherer S, Dunckley H, Trejaut J, Chapman J, et al Survival of DNA HLA-DR typed and matched cadaver kidney transplants. The Collaborative Transplant Study. Lancet. 1991; 338: 461–463. 1678443 10.1016/0140-6736(91)90540-6

[60] Ohno S. Immunological aspects of Behcet's and Vogt-Koyanagi-Harada's diseases. Trans Ophthalmol Soc U K. 1981; 101 (Pt 3): 335–341. 6820735

[61] Ikeda N, Kojima H, Nishikawa M, Hayashi K, Futagami T, Tsujino T, et al Determination of HLA-A, -C, -B, -DRB1 allele and haplotype frequency in Japanese population based on family study. Tissue Antigens. 2015; 85: 252–259. 10.1111/tan.12536 25789826 PMC5054903

[62] Numaga J, Matsuki K, Tokunaga K, Juji T, Mochizuki M. Analysis of human leukocyte antigen HLA-DR beta amino acid sequence in Vogt-Koyanagi-Harada syndrome. Invest Ophthalmol Vis Sci. 1991; 32: 1958–1961. 2055689

[63] Islam SM, Numaga J, Fujino Y, Hirata R, Matsuki K, Maeda H, et al HLA class II genes in Vogt-Koyanagi-Harada disease. Invest Ophthalmol Vis Sci. 1994; 35: 3890–3896. 7928186

[64] Shindo Y, Ohno S, Yamamoto T, Nakamura S, Inoko H. Complete association of the HLA-DRB1*04 and -DQB1*04 alleles with Vogt-Koyanagi-Harada's disease. Hum Immunol. 1994; 39: 169–176. 8026985 10.1016/0198-8859(94)90257-7

[65] Sugita S, Takase H, Taguchi C, Imai Y, Kamoi K, Kawaguchi T, et al Ocular infiltrating CD4+ T cells from patients with Vogt-Koyanagi-Harada disease recognize human melanocyte antigens. Invest Ophthalmol Vis Sci. 2006; 47: 2547–2554. 16723469 10.1167/iovs.05-1547

[66] Sugita S, Takase H, Kawaguchi T, Taguchi C, Mochizuki M. Cross-reaction between tyrosinase peptides and cytomegalovirus antigen by T cells from patients with Vogt-Koyanagi-Harada disease. Int Ophthalmol. 2007; 27: 87–95. 17253112 10.1007/s10792-006-9020-y

[67] Kawabata Y, Ikegami H, Awata T, Imagawa A, Maruyama T, Kawasaki E, et al Differential association of HLA with three subtypes of type 1 diabetes: fulminant, slowly progressive and acute-onset. Diabetologia. 2009; 52: 2513–2521. 10.1007/s00125-009-1539-9 19812988

[68] Mitsunaga S, Suzuki Y, Kuwana M, Sato S, Kaneko Y, Homma Y, et al Associations between six classical HLA loci and rheumatoid arthritis: a comprehensive analysis. Tissue Antigens. 2012; 80: 16–25. 10.1111/j.1399-0039.2012.01872.x 22471586

[69] Dorak MT, Tang J, Tang S, Penman-Aguilar A, Coutinho RA, Goedert JJ, et al Influence of human leukocyte antigen-B22 alleles on the course of human immunodeficiency virus type 1 infection in 3 cohorts of white men. J Infect Dis. 2003; 188: 856–863. 12964117 10.1086/378071

[70] Hendel H, Caillat-Zucman S, Lebuanec H, Carrington M, O'Brien S, Andrieu JM, et al New class I and II HLA alleles strongly associated with opposite patterns of progression to AIDS. J Immunol. 1999; 162: 6942–6946. 10352317

[71] Watanabe H, Okumura M, Hirayama K, Sasazuki T. HLA-Bw54-DR4-DRw53-DQw4 haplotype controls nonresponsiveness to hepatitis-B surface antigen via CD8-positive suppressor T cells. Tissue Antigens. 1990; 36: 69–74. 1702907 10.1111/j.1399-0039.1990.tb01802.x

[72] Usuku K, Sonoda S, Osame M, Yashiki S, Takahashi K, Matsumoto M, et al HLA haplotype-linked high immune responsiveness against HTLV-I in HTLV-I-associated myelopathy: comparison with adult T-cell leukemia/lymphoma. Ann Neurol. 1988; 23 Suppl: S143–150. 2894806 10.1002/ana.410230733

[73] Kuzushita N, Hayashi N, Moribe T, Katayama K, Kanto T, Nakatani S, et al Influence of HLA haplotypes on the clinical courses of individuals infected with hepatitis C virus. Hepatology. 1998; 27: 240–244. 9425943 10.1002/hep.510270136

[74] Kikuchi I, Ueda A, Mihara K, Miyanaga O, Machidori H, Ishikawa E, et al The effect of HLA alleles on response to interferon therapy in patients with chronic hepatitis C. Eur J Gastroenterol Hepatol. 1998; 10: 859–863. 9831409 10.1097/00042737-199810000-00009

[75] Minoda H, Sakai J, Sugiura M, Imai S, Osato T, Usui M. High inducibility of Epstein-Barr virus replication in B lymphocytes in Vogt-Koyanagi-Harada disease. Nippon Ganka Gakkai Zasshi. 1999; 103: 289–296. 10339973

[76] Raulet DH, Vance RE, McMahon CW. Regulation of the natural killer cell receptor repertoire. Annu Rev Immunol. 2001; 19: 291–330. 11244039 10.1146/annurev.immunol.19.1.291

[77] Yokoyama WM, Kim S. Licensing of natural killer cells by self-major histocompatibility complex class I. Immunol Rev. 2006; 214: 143–154. 17100882 10.1111/j.1600-065X.2006.00458.x

[78] Raulet DH, Vance RE. Self-tolerance of natural killer cells. Nat Rev Immunol. 2006; 6: 520–531. 16799471 10.1038/nri1863

[79] Martin MP, Qi Y, Gao X, Yamada E, Martin JN, Pereyra F, et al Innate partnership of HLA-B and KIR3DL1 subtypes against HIV-1. Nat Genet. 2007; 39: 733–740. 17496894 10.1038/ng2035 PMC4135476

[80] Yeh S, Karne NK, Kerkar SP, Heller CK, Palmer DC, Johnson LA, et al Ocular and systemic autoimmunity after successful tumor-infiltrating lymphocyte immunotherapy for recurrent, metastatic melanoma. Ophthalmology. 2009; 116: 981–989.e981. 10.1016/j.ophtha.2008.12.004 19410956 PMC2715843

[81] Crosson JN, Laird PW, Debiec M, Bergstrom CS, Lawson DH, Yeh S. Vogt-Koyanagi-Harada-like syndrome after CTLA-4 inhibition with ipilimumab for metastatic melanoma. J Immunother. 2015; 38: 80–84. 10.1097/CJI.0000000000000066 25658618 PMC4564122

[82] Wong RK, Lee JK, Huang JJ. Bilateral drug (ipilimumab)-induced vitritis, choroiditis, and serous retinal detachments suggestive of vogt-koyanagi-harada syndrome. Retin Cases Brief Rep. 2012; 6: 423–426. 10.1097/ICB.0b013e31824f7130 25389947

